# Clozapine and Regulatory Inertia: Revisiting Evidence, Risks, and Reform

**DOI:** 10.3390/healthcare13141668

**Published:** 2025-07-10

**Authors:** Carlos De las Cuevas

**Affiliations:** Department of Internal Medicine, Dermatology and Psychiatry and Instituto Universitario de Neurociencia (IUNE), Universidad de La Laguna, 38200 La Laguna, Spain; ccuevas@ull.edu.es

**Keywords:** clozapine, treatment-resistant schizophrenia, underutilization, regulatory barriers

## Abstract

In the United States, the Clozapine Risk Evaluation and Mitigation Strategy (REMS) program was implemented to ensure safe prescription and monitoring; however, its administrative complexity has often resulted in unintended barriers to access. Clozapine remains the most effective antipsychotic for treatment-resistant schizophrenia (TRS), yet its use continues to be constrained by outdated regulatory frameworks, cultural inertia, and clinical hesitancy. This perspective article revisits the pharmacokinetic foundations of clozapine, re-examines its association with fatal outcomes, and critiques the persistence of obsolete monitoring systems such as the U.S. REMS program. Drawing on recent consensus publications endorsed by over 120 international clozapine experts, this article outlines the proposed changes to the U.S. prescription information and contextualizes them within broader global practices. This article argues that many barriers to clozapine use stem not from evidence, but from regulatory conservatism and the perpetuation of clinical myths. The dismantling of the REMS program in early 2025 represents a pivotal moment, yet further reforms are urgently needed to align regulatory guidance with contemporary science. Ultimately, this article is a call to rediscover the clinical value of clozapine and to translate decades of knowledge into regulatory and clinical action.

## 1. Introduction

Clozapine, although being the only antipsychotic with proven efficacy for treatment-resistant schizophrenia (TRS), remains underutilized across most countries [1]. This paradox persists despite over four decades of accumulated clinical experience and robust evidence demonstrating its superior efficacy compared to other antipsychotics, especially in reducing suicidality and improving outcomes in TRS [2,3].

Multiple factors contribute to this underuse, including clinician hesitancy, outdated safety concerns, and a regulatory framework that has not adapted to emerging scientific insights [4,5]. Although the U.S. Food and Drug Administration (FDA) took an important step in 2025 by eliminating the centralized Clozapine Risk Evaluation and Mitigation Strategy (REMS) program [6], key aspects of the official prescription information (labeling) remain misaligned with current pharmacological understanding and global best practices.

Scientific evidence now points to a number of preventable causes of clozapine-related fatalities, such as pharmacokinetic mismanagement during infections, dose escalation without accounting for inflammatory markers, or oversedation leading to aspiration pneumonia [7,8,9]. Importantly, these risks are not intrinsic to clozapine itself but are exacerbated by clinical and regulatory mismanagement.

International comparisons further underscore these discrepancies. Countries such as Finland and Canada, where guidelines reflect contemporary pharmacokinetic and pharmacovigilance knowledge, report better clozapine utilization rates and outcomes without an increased adverse event burden [10,11].

This article synthesizes recent expert consensus publications—endorsed by over 120 international researchers—which collectively propose updates to the U.S. clozapine label, including ancestry-informed dosing, CRP-guided monitoring, and revised boxed warnings [12,13]. It argues that current barriers to clozapine use are no longer justified by evidence, and that harmonizing clinical and regulatory practice with contemporary science is both necessary and overdue.

This perspective was prompted by a series of publications led by Dr. José de Leon, whose extensive contributions have redefined the pharmacokinetic interpretation of clozapine and offered pragmatic recommendations for safer, more individualized use. His work has been instrumental in catalyzing a renewed international dialog on clozapine reform, providing both the scientific foundation and the ethical imperative for the present article.

## 2. Revisiting Clozapine Pharmacokinetics: Insights from the Literature

Part I of the two-part proposal to the FDA [12] presents a rigorous review of over 400 scientific articles addressing the pharmacokinetics and dosing considerations of clozapine. Among the central findings is the persistent mischaracterization of clozapine metabolism in the current U.S. package insert. Contrary to existing labeling, CYP1A2—not CYP2D6—is the primary enzyme responsible for clozapine metabolism. The current insert’s emphasis on poor metabolizers of CYP2D6 is misleading and clinically irrelevant.

Additionally, the document underscores the profound impact of systemic inflammation on clozapine plasma levels. During episodes of infection or inflammatory states, cytokine-mediated downregulation of CYP1A2 can result in a dangerous elevation of clozapine concentrations. Yet, these real-world scenarios are absent from the label.

Perhaps most striking is the need for ancestry-specific dosing guidance. Patients of Asian, Indigenous American, or other non-European ancestry typically require lower doses to achieve therapeutic levels, a fact consistently documented but unaddressed in current regulatory documents. This omission has profound implications, contributing to both under-treatment and adverse effects in these populations.

The article proposes clear, actionable revisions to the U.S. label, including the removal of inaccurate warnings, the addition of dosing tables by ancestry and inflammation status, and the acknowledgment of systemic factors that affect drug metabolism. These updates would bring the label in line with modern pharmacological understanding and reduce preventable harm (Table 1).

## 3. Clozapine-Associated Fatalities: Misaligned Regulatory Priorities

In Part II [13], the focus shifts from pharmacokinetics to pharmacovigilance. Drawing on data from the U.S. reports within the WHO global pharmacovigilance database, VigiBase, the authors reveal a striking disconnect between the labeled warnings and actual fatal outcomes.

Neutropenia, while historically emphasized, accounts for just a small minority of deaths (2.3%). In contrast, pneumonia and other infection-related events represent the leading causes of clozapine-associated mortality. These events are often preventable and frequently linked to mechanisms such as sedation-induced aspiration or unrecognized immunosuppression. Yet, they remain unmentioned in the U.S. boxed warnings label.

Even more troubling is the systemic disregard for early clinical warning signs. For example, elevated white blood cell counts—commonly present during infection—are often misinterpreted as signs of safety rather than red flags for emergent toxicity. A more nuanced label would help clinicians recognize these dynamic signals and intervene earlier.

The authors advocate for a restructuring of the boxed warnings to reflect data-driven priorities. Rather than continuing to emphasize agranulocytosis as the singular critical threat, the updated label should include infection-related risks and guidance on managing clozapine during febrile or inflammatory episodes. Such a change would not only improve patient safety but also align practice with contemporary evidence.

While we argue for a shift in regulatory focus, it is important to acknowledge the historical rationale behind prior caution. Early fatalities due to agranulocytosis understandably shaped regulatory conservatism and reinforced stringent monitoring protocols. However, advances in hematological surveillance, a better understanding of pharmacokinetics, and emerging real-world evidence now justify a reassessment of this paradigm. Continued adherence to outdated safety frameworks risks perpetuating harm through underutilization rather than preventing it.

## 4. The Clozapine REMS Program: A Case Study in Regulatory Inertia

The Clozapine Risk Evaluation and Mitigation Strategy (REMS) program, instituted by the FDA in 2015 [14], was designed to monitor and mitigate the risk of severe neutropenia associated with clozapine use. This centralized system required prescribers and pharmacies to be certified and mandated the regular reporting of patients’ absolute neutrophil count (ANC) before dispensing the medication. While the intention was to enhance patient safety, over time, the REMS program became a significant barrier to clozapine access, leading to treatment delays and interruptions.

In November 2024, a joint meeting of the FDA’s Drug Safety and Risk Management Advisory Committee and Psychopharmacologic Drugs Advisory Committee convened to re-evaluate the necessity of the clozapine REMS program. The committees voted overwhelmingly (14–1) in favor of eliminating the REMS requirements, citing that the program posed unnecessary obstacles to patient care and did not significantly enhance safety outcomes. Testimonies from clinicians, patients, and caregivers highlighted the detrimental impact of REMS-related delays, including the exacerbation of symptoms, hospitalizations, and, in some cases, suicide attempts [15].

Following the advisory committee’s recommendation, the FDA announced on 24 February 2025 that it no longer expects prescribers, pharmacies, and patients to participate in the clozapine REMS program. The agency acknowledged that, although the risk of severe neutropenia remains, the REMS program is no longer necessary to ensure that the benefits of clozapine outweigh its risks. The FDA instructed manufacturers to submit modifications to eliminate the REMS requirements and update the prescription information accordingly [16,17].

Despite this regulatory shift, challenges persist. Reports indicate that some pharmacies and insurance providers continue to enforce outdated REMS protocols, leading to confusion and continued barriers to access. This lag in implementation underscores the complexities of regulatory change and the need for coordinated efforts to ensure that policy updates translate into practice effectively [17].

The evolution and eventual dismantling of the clozapine REMS program exemplify the broader theme of regulatory inertia addressed in this perspective. It highlights the critical importance of aligning regulatory frameworks with current scientific evidence and clinical realities to optimize patient care.

## 5. Clinical Myths and Cultural Resistance

Despite the robust data supporting clozapine’s efficacy and safety when used appropriately, its prescription is still hindered by entrenched myths and cultural resistance within the psychiatric profession. These myths include the belief that clozapine is excessively dangerous, that the burden of hematologic monitoring outweighs its benefits, and that it should be reserved only for “last resort” cases [18].

Several studies have shown that psychiatrists often overestimate the incidence of agranulocytosis while underestimating the protective effect of clozapine against suicide and rehospitalization. In a systematic review, Tungaraza and Farooq [19] highlighted that the primary barriers to clozapine prescription were clinician attitudes, lack of experience, and institutional inertia—not clinical contraindications.

This disconnect is further amplified by medical training programs that inadequately expose young clinicians to clozapine prescription. As a result, the cycle of avoidance perpetuates itself. The label’s outdated warnings reinforce fears, while the REMS infrastructure historically magnified them. Unless addressed through education, reform, and leadership, these cultural barriers will continue to undermine the scientific evidence base.

## 6. A Global Call to Reframe the Label

The implications of clozapine underutilization are not confined to the United States. Package inserts and national guidelines around the world often mirror the FDA’s outdated framework or adopt even more conservative stances [20]. While countries like Finland and Canada have made strides in promoting earlier clozapine initiation and broader accessibility, many regulatory agencies continue to echo a pharmacovigilance model frozen in the 1990s.

An international consensus is needed—not merely to revise the U.S. label, but to set a new global benchmark for clozapine guidance. This includes eliminating disproven contraindications, clarifying dose adjustments by ancestry and inflammation, and emphasizing infection monitoring [21].

Such a change must also be institutionalized in psychiatric curricula, continuing medical education, and the protocols of pharmacovigilance agencies. The reinterpretation of clozapine is not merely an academic issue—it is a global public health imperative.

## 7. International Benchmarks: Lessons from Finland and Canada

While clozapine remains underutilized in many parts of the world, Finland and Canada exemplify how systematic approaches can optimize its use and improve outcomes for patients with treatment-resistant schizophrenia (TRS).

In Finland, clozapine has been embraced earlier and more frequently than in most other countries. Nationwide studies show that Finland has among the highest clozapine usage rates globally, with up to 20% of patients with schizophrenia being treated with the drug. Longitudinal cohort studies have demonstrated that the early introduction of clozapine—particularly after the first failed trial with another antipsychotic—is associated with better long-term outcomes, including lower rates of hospitalization and all-cause mortality [22].

Canada, while not reaching Finland’s usage levels, offers another instructive model. Canadian clinical guidelines explicitly recommend clozapine for patients who fail to respond adequately to two different antipsychotic agents [23]. However, studies have shown that there are still delays in clozapine initiation, often due to clinician hesitancy, logistical concerns about monitoring, and misconceptions regarding its safety profile. To address this, Canada has launched various educational- and systems-level initiatives to support clozapine prescribers. For example, integration with electronic health records and centralized hematologic monitoring services have helped streamline the process.

Both countries underscore the importance of infrastructure, education, and culture. Finland exemplifies a system where clozapine is viewed as a core treatment, not a last resort. Canada highlights the impact of national guidelines and targeted interventions in bridging the gap between evidence and practice. These international examples provide compelling evidence that better clozapine utilization is not only possible—it is already happening.

## 8. Implications for Clinical Practice and Regulatory Policies

Moving forward, rediscovering clozapine must involve not only reflection but also specific actions. These actions should span regulatory reform, clinical education, and systemic advocacy to address the practical barriers that continue to limit the drug’s potential.

(1) **Regulatory Revision**: Agencies such as the FDA and EMA must commit to revising the clozapine package insert based on current evidence. This includes adding warnings about infection-related mortality, updating metabolic pathway guidance, and incorporating dosing recommendations sensitive to ancestry and inflammatory status.
(2) **Clinical Guidelines and Training**: National psychiatric associations should issue updated clinical practice guidelines that incorporate evidence-based strategies for clozapine initiation, titration, and monitoring. Educational initiatives must be launched to train early-career psychiatrists and primary-care providers in the safe and effective use of clozapine.
(3) **Pharmacovigilance Integration**: Real-world data from VigiBase and other pharmacovigilance systems should be integrated into regulatory frameworks as dynamic sources of safety information, not merely as retrospective confirmations.
(4) **Implementation Science Approach**: Policymakers should apply implementation science methodologies to identify barriers and facilitators to clozapine use. This may include streamlining laboratory monitoring logistics, improving electronic health record alerts, and reducing stigma through targeted campaigns.
(5) **Global Benchmarks**: Countries such as Finland and Canada offer instructive examples of how clozapine can be safely deployed with higher rates of use and better patient outcomes. Learning from their experiences can guide U.S. and global improvements.

The future direction is clear: align policy with data, reshape education to match evidence, and confront regulatory inertia with the same rigor we apply to scientific inquiry. Only then will clozapine fulfill the promise it has held for decades.

We hope this perspective article will serve as both a summary and a catalyst: a summary of the compelling scientific case for change, and a catalyst for regulators, clinicians, and public health leaders to do what must be done: revise the package insert, align it with the evidence, and remove unnecessary barriers to the use of one of psychiatry’s most effective treatments.

It is time to rediscover clozapine. And this time, to not forget it again.

## 9. Implementation Challenges

Beyond the regulatory landscape, additional practical obstacles persist in day-to-day implementation. Despite growing consensus on the safety and clinical value of clozapine, its implementation continues to be hindered by multiple systemic barriers that extend beyond regulatory frameworks. These obstacles often manifest not in scientific debate, but in the everyday realities of healthcare delivery.

One persistent challenge is the logistical difficulty of initiating and maintaining clozapine in rural or underserved regions. In these settings, access to laboratory services for mandatory blood monitoring may be limited or delayed, dissuading both clinicians and patients. While point-of-care testing and digital health platforms offer potential solutions, they remain underutilized or unavailable in many healthcare systems [24].

Another critical issue is the fragmentation between primary care and specialized mental health services [25]. Clozapine initiation typically requires psychiatric oversight, yet ongoing monitoring may fall to general practitioners unfamiliar with the drug’s nuanced management. This lack of coordination can lead to delays, discontinuation, or underdosing, especially in systems where shared care protocols are poorly defined or absent.

Moreover, electronic prescribing systems and safety alerts, although well-intentioned, can inadvertently become restrictive [26]. Automated warnings or rigid dose thresholds embedded in software may prevent flexible titration or rapid response to clinical deterioration. In some jurisdictions, such systems have led to the denial of dispensing based on outdated contraindications or conservative algorithms that contradict current expert consensus.

Addressing these barriers requires not only updated guidelines, but also structural and technological reforms that empower clinicians to prescribe clozapine safely and confidently. Without attention to these implementation issues, even the most well-conceived regulatory revisions may fail to translate into improved patient outcomes.

## 10. Conclusions: Listening to the Forgotten Evidence

Clozapine is not merely a pharmacological tool—it represents a litmus test for how effectively our healthcare systems reconcile evidence, safety, and equitable access. Decades of cumulative data have established its unmatched efficacy in treatment-resistant schizophrenia, its specific protective effects against suicide, and a clearer, more nuanced understanding of its true risks. This accumulated knowledge demands a fundamental reassessment of how clozapine is regulated, prescribed, and taught across health systems [12,13].

Yet despite this clarity, institutional inertia persists. Regulatory guidance remains outdated in many jurisdictions. Medical curricula underrepresent its value. And patients are frequently delayed or denied access—not due to scientific uncertainty, but because of outdated fears, logistical burdens, and systemic hesitation [20].

- Pharmacovigilance data, particularly from global databases such as VigiBase, have been instrumental in shifting this paradigm [27]. These real-world safety insights have shown that risks once considered paramount—such as agranulocytosis—are now well-managed under current monitoring protocols, while other underrecognized threats—such as fatal pneumonia and cardiovascular events—deserve greater attention [28,29]. Continued post-marketing surveillance must inform updates to product labeling, prescribing practices, and educational strategies.

It is time to change course. Clozapine, as both a molecule and metaphor, challenges us to listen—not only to the peer-reviewed literature, but to the silenced experiences of those who were never offered it, who relapsed while waiting for it, or who died as outdated bureaucratic processes delayed access to it [30,31].

Progress in medicine is not only about discovering the new—it is about having the courage to rediscover the truth in what we already know, and the resolve to act upon it.

## Figures and Tables

**Table 1:** Proposed revisions to the U.S. clozapine prescription information.

Proposed Changes	Key Recommendations/Notes
**Pharmacology**	
CYP1A2 is the main metabolic pathway	- Women generally require lower doses because estrogens reduce the activity of this enzyme. - Smokers typically require higher doses because components of tobacco smoke increase enzyme activity. - Individuals of Asian or Indigenous American ancestry may require approximately half the usual dose due to lower enzymatic activity. - Obesity may also reduce enzyme activity, possibly requiring dose adjustments.
Other metabolic pathways	- At higher clozapine concentrations, CYP3A4 may become more relevant and could contribute to myocarditis. - Valproic acid may enhance clozapine clearance via glucuronidation.
Excretion and renal function	- Renal elimination of metabolites is clinically relevant. - In older adults, reduced renal function may justify a one-third dose reduction.
Drug monitoring and interactions	- Steady-state and trough clozapine levels should be measured. - Drug interaction lists should include pharmacokinetic, central, and peripheral interactions. - Updated guidance is needed for interactions with antidepressants, antibiotics, anti-seizure medications, benzodiazepines, and lithium.
Inflammation and infection	- Systemic inflammation (e.g., infections) reduces CYP1A2 activity, raising clozapine levels. - With markedly elevated CRP, the clozapine dose should be reduced by two-thirds. - Mild or no CRP elevation usually leads to only modest increases in clozapine levels.
Dosing strategy	- Gradual and individualized titration is essential. - Monitoring CRP during titration is recommended.
**Pharmacovigilance**	
Agranulocytosis monitoring	- Fatal risk is minimal with regular blood monitoring. - The centralized ANC database in the U.S. is no longer necessary. - Weekly monitoring during the first 18 weeks is generally sufficient. - Many low neutrophil counts are not clozapine-induced.
Pneumonia and infections	- Leading cause of death among clozapine-treated patients. - Data show 218 deaths from neutropenia vs. 1270 from infections. - Aspiration pneumonia (30%+) can be prevented via dose minimization, reduced sedation, and adjusting interacting drugs. - Non-aspiration infections may be linked to schizophrenia itself. - Pneumonia risk increases with excessive clozapine levels. - Rapid titration may cause eosinophilic pneumonia in rare cases.
Myocardial infarction (MI)	- Possibly the second most common cause of clozapine-related death. - May relate to TRS characteristics and high doses. - MI during high-dose clozapine treatment may increase mortality.
Age and sex variation in fatal ADRs	- Pneumonia is most frequent in those over 45. - Pulmonary embolism may dominate in younger women. - Suicide remains a leading concern in adolescents and young adult males.
